# Optimisation of a murine splenocyte mycobacterial growth inhibition assay using virulent *Mycobacterium tuberculosis*

**DOI:** 10.1038/s41598-017-02116-1

**Published:** 2017-06-06

**Authors:** Christina Jensen, Line Lindebo Holm, Erik Svensson, Claus Aagaard, Morten Ruhwald

**Affiliations:** 1 Department of Infectious Disease Immunology, Statens Serum Institut, Copenhagen, Denmark; 2 Copenhagen University Hospitals, Hvidovre, Copenhagen Denmark; 3 International Reference Laboratory of Mycobacteriology, Statens Serum Institut, Copenhagen, Denmark

**Keywords:** Antimicrobial responses, Cytokines, Pathogens, Protein vaccines, Tuberculosis

## Abstract

In the absence of a validated correlate of protection or robust animal models for human tuberculosis, Mycobacterial growth inhibition assays (MGIAs) aim to assess vaccines ability to inhibit mycobacterial growth *in-vitro*. We optimised a reproducible murine splenocyte MGIA based on *in-vitro* infection with ﻿virulent *Mycobacterium tuberculosis* (*M.tb)* Erdman. We identified splenocyte viability as a problem in state-of-art MGIA protocols, which can be improved by simple changes in culture conditions (viability increase from 21% to 46% at last day of culture). The growth inhibitory potential in mice immunised with either BCG, H56:CAF01 or H56:CAF01 administered side-by-side with BCG was significantly better compared to placebo in all groups (0.3 log_10_ CFU [±0.2, p = 0.049], 0.5 [±0.2, p = 0.016] and 0.6 [±0.1, p = 0.0007], respectively) corresponding to the levels of *in-vivo* protection. Unexpectedly the CAF01 adjuvant control group also induced significant growth inhibition of 0.3 log_10_ CFU (±0.2, p = 0.047). Finally, we explored vaccine-associated T cell effector functions. Despite presence of high levels of vaccine-specific T cells, we found no increase in CD4^+^ T cell number or cytokine expression profile, nor a difference in cytokine levels in the supernatant after four days culture with or without *M.tb*. Spontaneous IFN-γ release correlated with growth inhibition levels (p = 0.02), however the cellular source was not found.

## Introduction

Over the last two centuries, tuberculosis (TB) is estimated to have killed one billion people, and remains the world’s most lethal infectious disease^1^. The current tools for controlling TB are insufficient, and without new efficacious TB vaccines the WHO End TB strategic goals of a reduction of TB deaths by 95% and cases of TB disease by 90%, from 2015 and 2035 will not be met^2^. Drug-resistant TB is a growing threat to the epidemic, and since TB vaccines are expected to be equally effective against drug-sensitive and drug-resistant strains, vaccines are key to managing the spread of resistant strains.

A major roadblock in the development of new vaccines for TB is the absence of validated correlates of protection or robust animal models for human TB. Consequently, TB vaccine developers rely on large and expensive trials (more than 3,000 subjects) with long follow-up periods to generate proof of concept efficacy data^3, 4^. Therefore there is a relevant push for further research into animal models and correlates, as well as the integration of exploratory immunological projects nested in the clinical trials^5^.

Mycobacterial growth inhibition assays (MGIA) have been proposed as simple and unbiased tools to evaluate vaccine efficacy *in vitro* ^5, 6^. These assays study *in vitro* co-culturing of vaccine-induced cells and mycobacteria followed by a determination of the immune cells capacity of inhibiting mycobacterial growth. Several variations of human and murine MGIAs are described in the literature including assays based on human whole blood^7–11^ or PBMCs^8, 9, 12^ and murine assays based on splenocytes^13–15^ or pre-infected bone marrow derived macrophage target cells in splenocyte co-culture assays (BM/SP-MGIA)^16, 17^. The predominate organism used for both vaccination and *in vitro* challenge is BCG^6^.

Encouragingly, several of the murine MGIAs have demonstrated significant *in vitro* growth inhibition in a BCG vaccination model corresponding to *in vivo* protection in parallel challenge studies^14, 15, 17^. Within the last years, there has been a drive towards protocol harmonisation and standardisation in an otherwise heterogeneous field. In particular, a standardised murine MGIA based on direct co-culturing of mouse splenocytes with BCG has been proposed as a robust and simpler version of the BM/SP-MGIA^13, 15, 18^. This protocol was optimised and qualified with particular emphasis on multiplicity of infection (MOI) for low assay variability and widest window of growth inhibition^15^. However, it remains to be demonstrated that the underlying mechanism responsible for the observed growth inhibition is driven by vaccine-specific adaptive immunity, as well as essential assay parameters such as cell viability and T cell effector functions during the four-day culture are unknown^13–15^.

Therefore, we aimed to characterise and optimise a murine splenocyte MGIA to study the growth inhibitory potential of experimental TB vaccines *in vitro*. We based our assay on the current state-of-art protocol^15, 18^ and aimed to describe fundamental parameters and estimate variability of the assay. Under the assumptions that *Mycobacterium tuberculosis* (*M.tb)* is an intracellular pathogen *in vivo* and *in vitro* and that cellular immunity is essential for host control of infection, we focused on the adaptive immune responses. Instead of using BCG as the *in vitro* infectious organism as in the previously described murine splenocyte MGIAs^13, 15^, the virulent mycobacterial strain *M.tb* Erdman was used.

## Materials and Methods

### Animals

Six- to eight-weeks old female CB6F1 mice (BALB/c × C57BL/6, Envigo, Horst, Netherlands) rested 1-week were housed and handled in Biosafety Level 2 (BSL2) animal facilities at Statens Serum Institut, Denmark and were provided standard food and water *ad libitum*. The handling of mice was conducted in accordance with the regulations set forward by the national animal protection committee in compliance with European Community Directive 2010/63. In agreement with the Danish Animal Welfare Act all experimental methods including protocols involving animals were carried out in accordance with relevant guidelines and regulations. All protocols were reviewed prior to the start of the experiment by an independent ethical review board at Statens Serum Institut and approved to be in accordance with our license for animal experiments issued by The Animal Experiments Inspectorate (License no. 2014-15-2934-01065) under the Ministry on Environment and Food of Denmark.

### Immunisation

The mice were immunised subcutaneously (s.c.) three times at 2-week intervals with either Tris HCL buffer or CAF01 (dose 250 μg/50 μg (DDA/TDB)) alone or CAF01 mixed with 5 μg H56 protein, produced as previously described^19^. Positive control mice received a single dose 200 µl of 2.5 × 10^6^ Colony Forming Units (CFU)/ml BCG Danish 1331 (Statens Serum Institut).

When H56 was used as a BCG booster vaccine (H56:CAF01 side-by-side (SBS) with BCG), mice were vaccinated with 200 µl 2.5 × 10^6 ^CFU/ml BCG the first day and then with 0.1 μg H56 in CAF01 the next day, followed by two H56:CAF01 immunisations, 2-weeks apart. In the first vaccination round, mice were vaccinated with 100 μl BCG or H56:CAF01 s.c into the left and right side of the base of the tail. Unless specified otherwise, splenocytes were isolated one week after last immunisation.

### Cell culture optimisation

Single splenocyte suspensions were prepared by homogenisation through 100 μm cell strainers followed by washing in RPMI 1640 (Invitrogen) and adjustment to 5 × 10^6^ splenocytes per 600 μl in MGIA media. MGIA media were either standard media (RPMI-1640, 10% heat-inactivated FCS (Biochrom Gmbh) + 10 mM Hepes (Invitrogen) + 2 mM L-Glutamine (Invitrogen))^15^ or enriched media (standard media + 1 mM Natriumpyruvate (Invitrogen) + 1 × Non-essential amino acids (MP Biomedicals, LLC) + 5 × 10^−5 ^M 2-mercaptoethanol (Sigma-Aldrich)). The cell suspensions were cultured in 2 ml screw cap tubes (Sarstedt) on a 360° tube rotator (Intelli-Mixer Rm-2 L, ELMI) or in a rack at 37 °C for four days. At different time points, the splenocytes were counted with an automatic Nucelocounter^TM^ (Chemotec) or manually using Nigrosine. All cell work pre-*M.tb* infection was done in BSL2.

### Mycobacteria and culture conditions

For *in vitro* infection a frozen vial of *M.tb* Erdman (ATCC strain, grown in 7H9 broth stored at −80 °C) was thawed in a water bath followed by 5 minutes sonication. Any clumps were removed by three times of syringe aspiration. Mycobacterial suspensions for infection inoculum and BACTEC MGIT standards were prepared in enriched media by serial 10-fold dilutions. All work involving *M.tb* infected samples was done at BSL3.

### *In vitro* mycobacterial growth inhibition assay

*M.tb* Erdman was prepared in enriched media aiming at a concentration of 167 CFU/ml (unless specified otherwise). Within one hour from preparation, 300 μl mycobacterial suspension was added to 5 × 10^6^ splenocytes prepared in 300 μl enriched media (corresponding to an inoculum of 50 CFU per sample tube). *M.tb*-splenocyte co-cultures were incubated in a rack at 37 °C for four days followed by 10 min centrifugation at 12.000 rpm in a bench-top microcentrifuge. One-hundred μl supernatant was removed for multiplex cytokine assays and the remaining 500 μl were resuspended, added to a MGIT tube (BD Biosciences) and incubated until registered positive (BACTEC MGIT liquid culture system (BD Biosciences)). The resulting time to positivity (TTP) was converted to bacterial numbers (CFU) using a linear regression of a standard curve comprised of TTP values from inoculated *M.tb* Erdman 10-fold dilutions against CFUs obtained from plating aliquots of *M.tb* Erdman onto Middlebrook 7H11 agar plates (BD Biosciences). Direct-to-MGIT controls were included, defined as 50 CFU *M.tb* Erdman directly placed in the BACTEC MGIT system without any pre-incubation (at day 0). Data are presented as total number log_10 _CFUs per sample tube. To compare the growth inhibition between experiments, delta log_10 _CFU was calculated by subtracting the individual log_10 _CFU values in the immunised group from the mean of the control group.

For examination of intracellular growth, splenocyte-mycobacteria co-cultures were incubated for three hours, then treated with 0 or 100 μg/ml gentamicin (Gibno, Life Technologies) for one hour followed by three times wash and placement in the BACTEC MGIT system. Samples without splenocytes were cultured and treated in parallel without wash before TTP assessment.

### Intracellular cytokine staining assay

A total of 1–2 × 10^6^ splenocytes were stimulated *in vitro* in V-bottom 96-well plates at 37 °C in media containing anti-CD49d (1 μg/ml) and anti-CD28 (1 μg/ml) without antigen or in the presence of 2 μg/ml H56 protein for 1 hour, plus 6 hours in the presence of 10 μg/ml brefeldin A (Sigma-Aldrich), after which cells were maintained at 4 °C until staining.

Cells were stained for the surface markers using anti-CD4-BV786 (clone GK1.5; BD Biosciences), anti-CD44-FITC (clone IM7; eBioscience, USA) and anti-Fixable Viability Dye-APC-Cy7 (eBioscience, USA) before fixation and permeabilisation using Cytoperm/cytofix kit (BD Biosciences) as per manufacturer’s instructions, and subsequently stained for intracellular cytokines with anti-IFN-γ-PeCy7 (clone XMG1.2; eBioscience, USA), anti-TNF-α-Pe (clone MP6-XT22; eBioscience, USA) and anti-IL-2-APC (clone JES6-544; eBioscience, USA). Non-specific background cytokine values were determined for each combinatorial Boolean gate and subtracted. Gates for surface markers were based on fluorescence-minus-one controls. All flow cytometry analyses including Boolean analysis were performed with FlowJo Software v.10 (Tree Star, Ashland, OR, USA).

### Multiplex cytokine assay

The Proinflammatory panel 1 (Mouse) 7-plex cytokine assay (Meso Scala Discovery (MSD)) measuring IFN-γ, IL-1β, IL-6, KC/GRO, IL-10, IL-12p70 and TNF-α was performed according to the manufacturer’s instructions. The plates were read on the Sector Imager 2400 system (Meso Scala Discovery) and calculation of cytokine concentrations in unknown samples was determined by 4-parameter logistic non-linear regression analysis of the standard curve.

### Statistical analysis

Prism 6 software (Graphpad v6.05) was used for all statistical analyses. Mean values and parametric tests were used under the assumption that data are normally distributed. Unpaired two-tailed t-tests were used to compare control and vaccinated groups in the MGIAs. Cytokine levels detected with MSD were analysed using one-way ANOVA with Dunnett’s multiple comparisons test. Associations between growth inhibition and cytokine responses were measured using Spearman’s rank correlation coefficient. A p-value of p < 0.05 was considered significant. Statistically significant differences are marked by asterisks in figures and explained in the figure legends.

## Results

### Assay optimisation and fundamental parameters in the splenocyte MGIA

**Figure 1:**
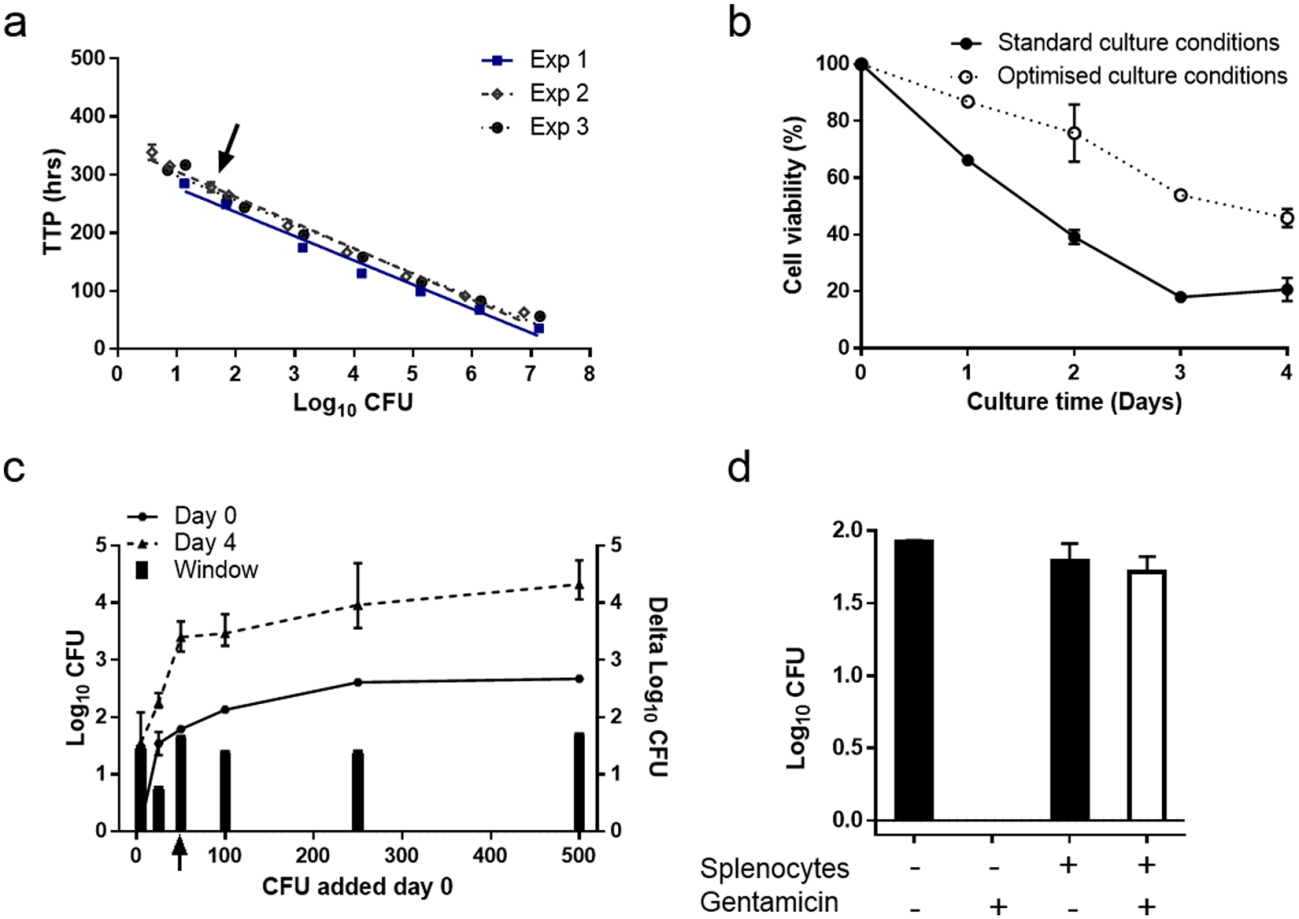
Assay optimisation and fundamental parameters in the splenocyte MGIA. (**a**) Results from three independent experiments (Exp 1, 2 and 3) in which serial 10-fold dilutions of *M.tb* Erdman were added MGIT tubes to produce a standard curve from which the time to positivity (TTP) could be related to inoculum size. Log_10_ colony forming units (CFU) were determined by plating aliquots of *M.tb* Erdman on agar plates. Error bars represent mean ± range of measurements done in duplicates. (**b**) Cell viability of naive splenocytes cultured in standard media with rotation (standard culture conditions) or in enriched media without rotation (optimised culture conditions). Error bars represent mean ± range of duplicates measured of splenocytes pooled from three naive mice. The results are representative of three independent experiments. Similar viability was confirmed by manual nigrosine count. (**c**) Splenocytes from individual naïve mice were co-cultured in four days with 5, 25, 50, 100, 250 or 500 CFU of *M.tb* Erdman under optimised culture conditions (Day 4). The inoculums were directly transferred to MGIT tubes at day 0 to generate a baseline (Day 0). Error bars represent mean ± range of measurements done in duplicates (Day 0). Error bars represent mean ± range of one mouse measured in triplicates (25, 50, 250 and 500 CFU) or in four replicates (5 and 100 CFU) (Day 4). The window was calculated by subtracting the mean values at Day 0 from Day 4. (**d**) Splenocytes from naïve mice were co-cultured for three hours with 50 CFU of *M.tb* Erdman followed by one hour of 0 or 100 μg/ml gentamicin treatment before they were inoculated in MGIT tubes. In parallel, samples without splenocytes were incubated. Error bars represent mean ± range of duplicates measured of splenocytes pooled from two mice.

The TTP was closely related to the number of CFUs per millilitre suspensions of *M.tb* Erdman. In three independent experiments, TTP values were found to be highly reproducible with a duplicate CV <6% in all titrations (5 to 1 × 10^7 ^CFU) (Fig. 1a). A low inter-assay variability was also detected, with a CV <6% across experiments in a concentration at 50 CFU per 600 µl culture media. Given the importance of viable functional effector T cells in TB vaccine immunology, we next focused on splenocyte viability during four-day culture. We initially described viability under standard culture conditions^15, 18^ wherein 5 × 10^6^ splenocytes from naive mice were isolated and cultured in standard media (RPMI, FCS, Hepes and L-Glutamine) and incubated at 37 °C with 360° rotation for four days^15, 18^. These conditions led to a rapid and substantial loss of viability with only mean 21% (range 17–25%) viable cells at day four (Fig. 1b). Enrichment of the culture media by addition of nutrients (Natriumpyruvate, Non-essential amino acids, and 2-mercaptoethanol) and incubation without rotation, increased cell viability at day four to 46% (43–49%) (Fig. 1b). Nutrient enrichment and no rotation were studied separately and demonstrated day four viability of 19% (17–21%) and 36% (35–37%), respectively (data not shown). Based on the assumption that viable splenocytes is an essential component in the study of vaccine-induced growth inhibition in this type of assay we advanced our experiments using nutrient enrichment without rotation.

We next focused on describing the co-culture of *M.tb* Erdman and vaccine naive splenocytes. Under the assumption that the lowest reproducible CFU inoculum demonstrates potential growth inhibition best^15^, we titrated the *M.tb* Erdman inoculum, determined the delta log growth from day zero to day four, and demonstrated a fairly consistent growth window of 1.6 log_10 _CFU with inoculum above 50 CFU per 600 µl culture media, which was used in the subsequent MGIA experiments (Fig. 1c). Finally, to verify that the mycobacteria grow intracellularly, splenocytes and mycobacteria were co-cultured for three hours to allow infection, followed by addition of 100 μg/ml gentamicin; an antibiotic which is not transported across the eukaryote cell membrane killing only extracellular bacteria. *M.tb* Erdman growth was unaffected by gentamicin in the extracellular environment when splenocytes were present, while there were no live mycobacteria in samples without splenocytes, indicating that the mycobacteria were indeed intracellular (Fig. 1d).

### Assay variability

**Figure 2:**
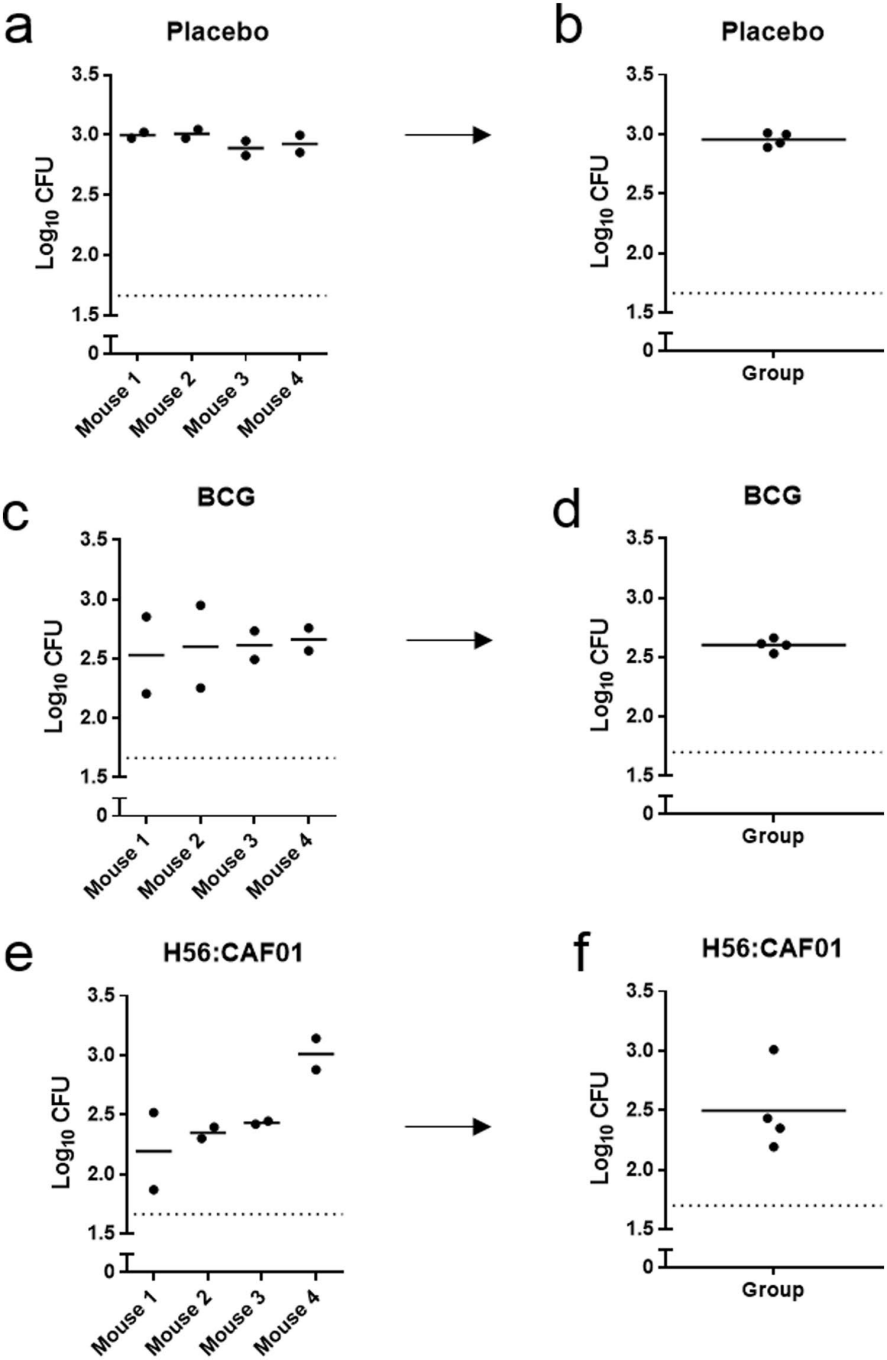
Assay variability. Groups of mice were immunised three times s.c. with H56 in CAF01 (**e**,**f**), one time with BCG (**c**,**d**) or placebo (Tris buffer) (**a**,**b**) with 2-week intervals. Splenocytes from individual mice (n = 4) were isolated one week after the last immunisation and co-cultured with 50 CFU of *M.tb* Erdman in the four days MGIA. The mean log_10 _CFU of measurements of individual mice done in duplicates (**a,c,e**), group means (**b,d,f**).

Next, we assessed the sample variability of our optimised MGIA. Groups of four mice were immunised with either BCG, H56:CAF01 or placebo, and splenocytes were assayed one week after the last immunisation (Fig. 2). We observed low within mouse duplicate variability in the placebo group (Coefficient of Variability (CV) 4%) (Fig. 2a) while duplicate variability was higher in the vaccinated mice (CV 19% and 21% in BCG (Fig. 2c) and H56:CAF01 (Fig. 2e), respectively). The variability within groups was also higher in the vaccinated groups with CV of 2%, 2% and 14% for placebo, BCG and H56:CAF01 (Fig. 2b,d and f), respectively.

### H56:CAF01 and BCG immunisation induced mycobacterial growth inhibition in murine splenocytes

**Figure 3:**
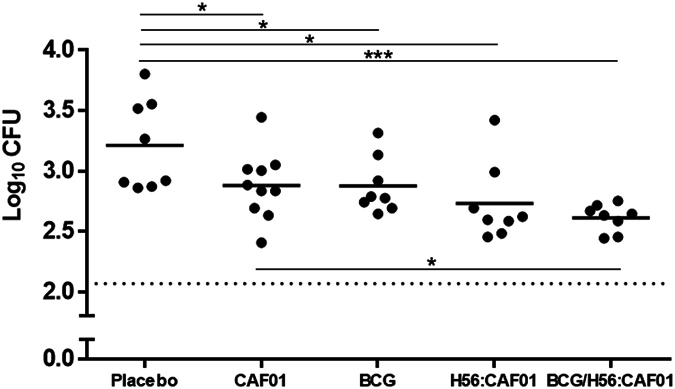
H56:CAF01 and BCG immunisation induced mycobacterial growth inhibition in murine splenocytes. Groups of mice were immunised three times s.c. with 2-week intervals with H56 in CAF01, H56:CAF01 SBS with BCG, adjuvant alone (CAF01) or Placebo (Tris buffer). At the same time as the first vaccination, a group of mice received a single dose BCG. Splenocytes were isolated one week after the last immunisation (5 weeks for BCG) and used in the MGIA. Solid lines represent the means of eight individual mice measured in duplicates (CAF01 n = 10). t-test, *p < 0.05; ***p < 0.001.

To determine whether the optimised MGIA could demonstrate growth inhibition *in vitro*, we selected a panel of experimental vaccines developed at Statens Serum Institut, which previously have shown protective in *in vivo* challenge experiments: BCG and H56:CAF01 (both ~1 log_10 _CFU protection^20^, and unpublished) and H56:CAF01 SBS with BCG (~1.3 log_10_, Aagaard unpublished and ref. 21). Groups of eight mice were immunised with the three vaccines and compared to a placebo and CAF01 adjuvant control (Fig. 3). Significant growth inhibition was observed in all groups, where H56:CAF01 SBS with BCG induced the strongest growth inhibition with a reduction of 0.6 (SEM ± 0.1) log_10 _CFU compared to the placebo group (p = 0.0007; t-test). Splenocytes from H56:CAF01 or BCG immunised mice induced growth inhibition with a reduction of 0.5 (±0.2, p = 0.016; t-test) and 0.3 (±0.2, p = 0.049; t-test) log_10 _CFU compared individually to placebo, respectively. Unexpectedly the CAF01 adjuvant control group mediated significant growth inhibition of 0.3 (±0.2, p = 0.047; t-test) log_10_ CFU on a level comparable to BCG. The application of one-way ANOVA with Dunnett’s adjustment for multiplicity resulted in significant growth inhibition in splenocytes from H56:CAF01 (p = 0.005) and H56:CAF01 SBS with BCG (p = 0.0005) vaccinated mice compared to the placebo group, supporting these vaccines as most potent in the system. Of note, we observed a larger within group variability also in the placebo group compared to the earlier variability assessment CV <12%.

### Mycobacterial growth inhibition is reproducible, allowing for comparison between experiments

**Figure 4:**
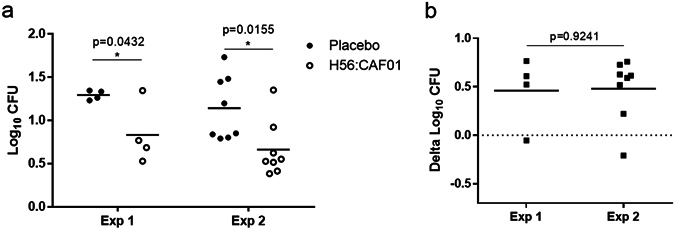
Mycobacterial growth inhibition is reproducible, allowing for comparison between experiments. Splenocytes from mice immunised three times s.c. with 2-week intervals with H56 in CAF01 or given placebo (Tris buffer) were isolated one week after the last immunisation and used in MGIA. Experiment (Exp) 1 and 2 represent two independent immunisation experiments. (**a**) Log_10 _CFU values after subtracting the direct-to-MGIT controls from the individual log_10 _CFU values. (**b**) Delta log_10 _CFU values represent the H56:CAF01 induced mycobacterial growth inhibition and were calculated by subtracting the individual log_10 _CFU values in the immunised H56:CAF01 groups from the mean of the respective control group (Placebo). Solid lines represent the mean Log_10 _CFU of 4 and 8 mice, for experiment 1 and 2 respectively, measured in duplicates. t-test, *p < 0.05.

We compared the between run variability in two separate H56:CAF01 vaccination experiments (data from Figs 2 and 3) after adjustment for different *M.tb* inocula (68 and 70 CFU) by subtraction of the log_10 _CFU growth in direct-to-MGIT controls from the individual sample values (Fig. 4a). H56:CAF01 immunisation induced a reproducible difference of 0.5 log_10 _CFU compared to the respective placebo group in both experiments (p = 0.92; t-test) (Fig. 4b) supporting the use of this subtraction method to allow comparison between experiments.

**Figure 5:**
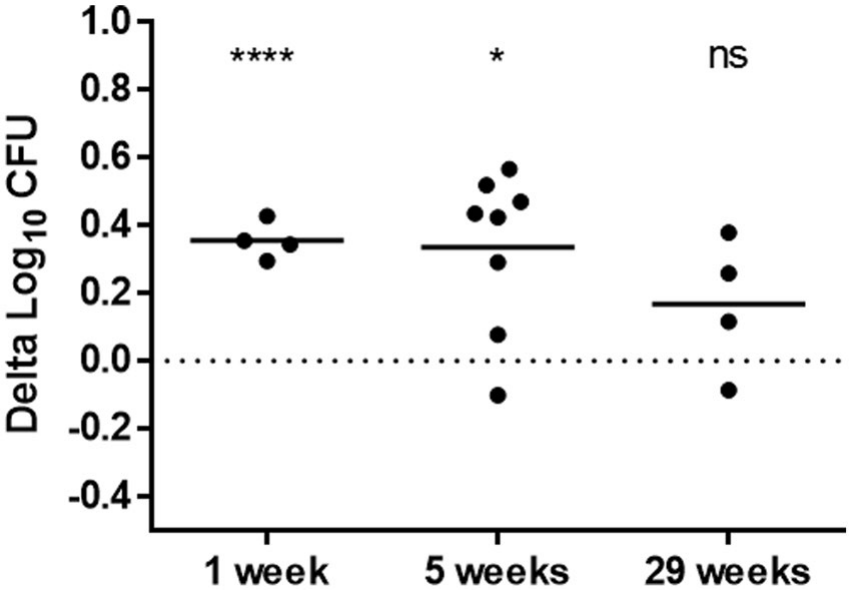
Kinetic in BCG-induced mycobacterial growth inhibition. Groups of mice were immunised with a single dose BCG or given Tris buffer (Placebo) three times s.c. with 2-week intervals. In different immunisation experiments, splenocytes were isolated 1 week, 5 weeks or 29 weeks after BCG immunisation and used in MGIA. Delta log_10 _CFU values represent the BCG-induced mycobacterial growth inhibition and were calculated by subtracting the individual log_10 _CFU values in the immunised BCG groups from the mean of the respective placebo group. Solid lines represent the mean delta log_10 _CFU of 4, 8 and 4 mice, for 1 week, 5 weeks or 29 weeks, respectively, measured in duplicates. t-test between the BCG group and the respective placebo group. *p < 0.05; ****p < 0.0001.

We then proceeded to investigate the temporal aspects of the vaccine-induced growth inhibition potential in splenocytes obtained 1, 5 and 29 weeks after BCG immunisation (Fig. 5). Significant growth inhibition in the BCG immunised versus the respective placebo group was observed 1 week (0.4 ± 0.04 log_10 _CFU; p < 0.0001; t test) and 5 weeks (0.3 ± 0.2 log_10 _CFU; p = 0.049; t-test) after BCG immunisation; however, interestingly this response was not detected 29 weeks after immunisation (0.2 ± 0.2 log_10 _CFU; p = 0.4; t-test) (Fig. 5). We also explored H56:CAF01 growth inhibition at 29 weeks, in line with the findings of BCG also this vaccine failed to control at the late time point (0.05 ± 0.3 log_10 _CFU; p = 0.9; t-test, data not shown).

### *In vitro* infection does not drive detectable change in T cell functionality

**Figure 6:**
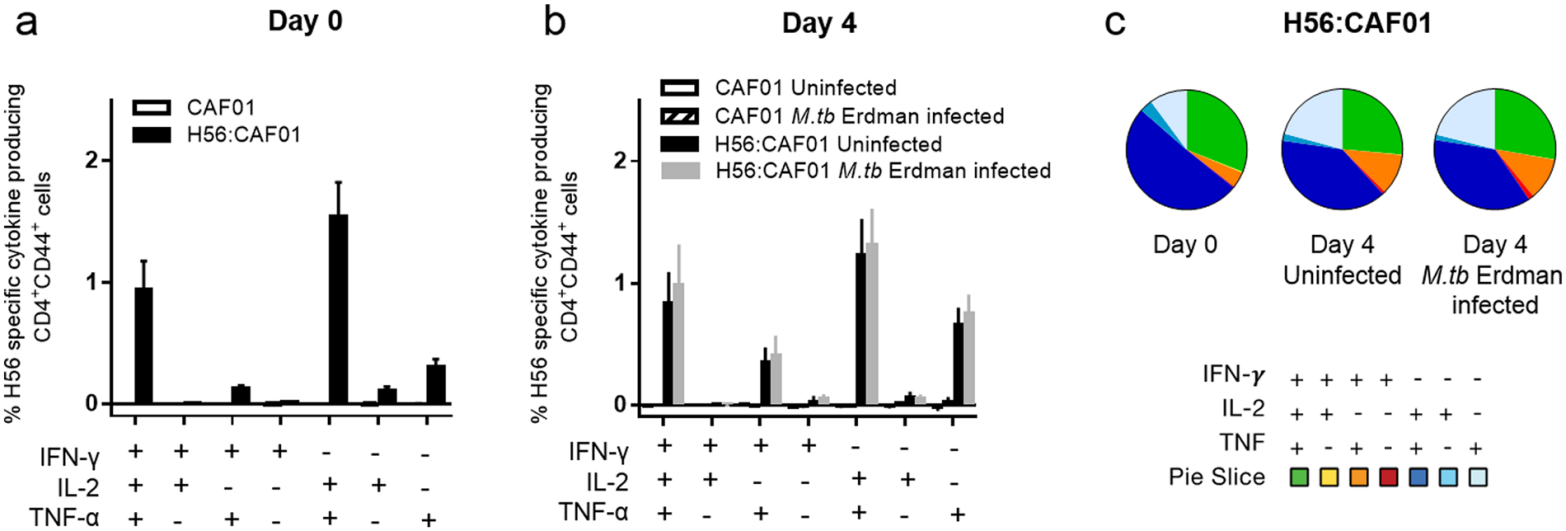
*In vitro* infection does not drive detectable change in T cell functionality. Groups of mice were immunised three times s.c. with 2-week intervals with H56 in CAF01 or adjuvant control (CAF01). One week after the last vaccination, splenocytes were isolated and used for intracellular cytokine analysis by flow cytometry at day 0 (**a**) or after four days culture with or without 50 CFU of *M.tb* Erdman (**b**). Splenocytes were stimulated with H56 *in vitro* before the frequencies of antigen-specific CD4^+^ cells (CD44^high^) producing IFN-γ, TNF-α and IL-2 were measured by gating for singlets, lymphocytes and live CD4^+^ cells. All possible combinations of cytokine expression were tabulated by Boolean gating analysis, and, after subtracting the background (non-stimulated) samples, the results for the seven combinations expressing at least one of the cytokines were shown. Bars represent mean + SEM of eight mice. (**c**) Pie charts over the polyfunctional CD4^+^ cells shown in Fig. 6a and b.

As an initial and crude control of the role of cellular immunity in the MGIA, we explored the mycobacterial growth in parallel cultures of live splenocytes from H56:CAF01 vaccinated mice compared to heat killed splenocytes from H56:CAF01 vaccinated mice (20 minutes at 60 °C) and found no indication of growth inhibition in the heat killed culture (data not shown). Therefore, under the hypothesis that MGIA measures a vaccine-specific T cell dependent mechanism, we investigated IFN-γ, TNF-α and IL-2 expression in CD4^+^ T cells following H56 stimulation in splenocytes from H56:CAF01 immunised mice using intracellular stain flow cytometry before and after four days culture with or without *M.tb* Erdman infection. In agreement with the literature^19^, H56:CAF01 induced a CD4^+^ T cell profile dominated by TNF-α^+^IL-2^+^, and IFN-γ^+^TNF-α^+^IL-2^+^ polyfunctional CD4^+^ T cells (Fig. 6a), which did not change on day four (Fig. 6b and c). T here was no indication of a change in frequency or phenotype of the CD4^+^ T cell population on day four comparing *M.tb* Erdman-infected and uninfected splenocyte-cultures (Fig. 6b and c).

### Association between polyfunctional T cells and mycobacterial growth inhibition

**Figure 7:**
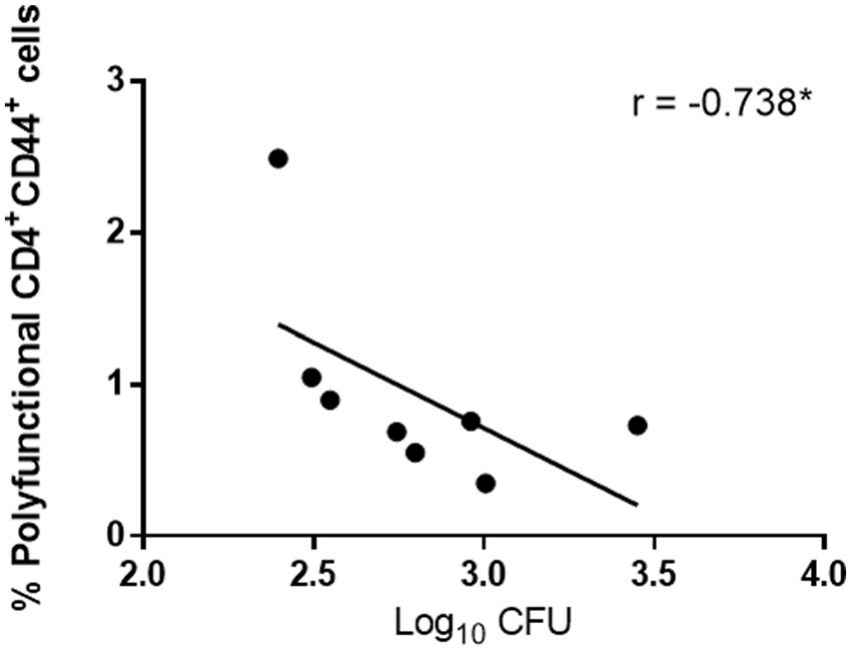
Association between polyfunctional T cells and mycobacterial growth inhibition. Scatter plots of the frequency of IFN-γ^+^TNF-α^+^IL-2^+^polyfunctional CD4^+^ cells at day 0 from the experiment shown in Fig. 6a versus H56:CAF01 induced growth inhibition data from the same experiment. Spearman’s rank *p < 0.05.

Next, we investigated whether the demonstrated vaccine-induced growth inhibition correlate with the cytokine flavour of the vaccine-induced CD4^+^ T cells at day zero in splenocytes from a separate experiment comparing responses in groups of eight H56:CAF01 and CAF01 control vaccinated mice. We observed a significant inverse correlation between IFN-γ^+^TNF-α^+^IL-2^+^ polyfunctional CD4^+^ T cell frequency and log_10 _CFU (Spearman r = −0.738; p = 0.046; Fig. 7) in the H56:CAF01 group, however, the data suggests that this association is driven by one outlier with the highest number of polyfunctional T cells, which after exclusion rendered the slope null. We repeated this exercise for all IFN-γ positive and TNF-α^+^IL-2^+^ T cells identifying a similar weak association where the slope seemingly was driven by the same outlier (data not shown).

### Cytokine release associated with vaccination but not infection

**Figure 8:**
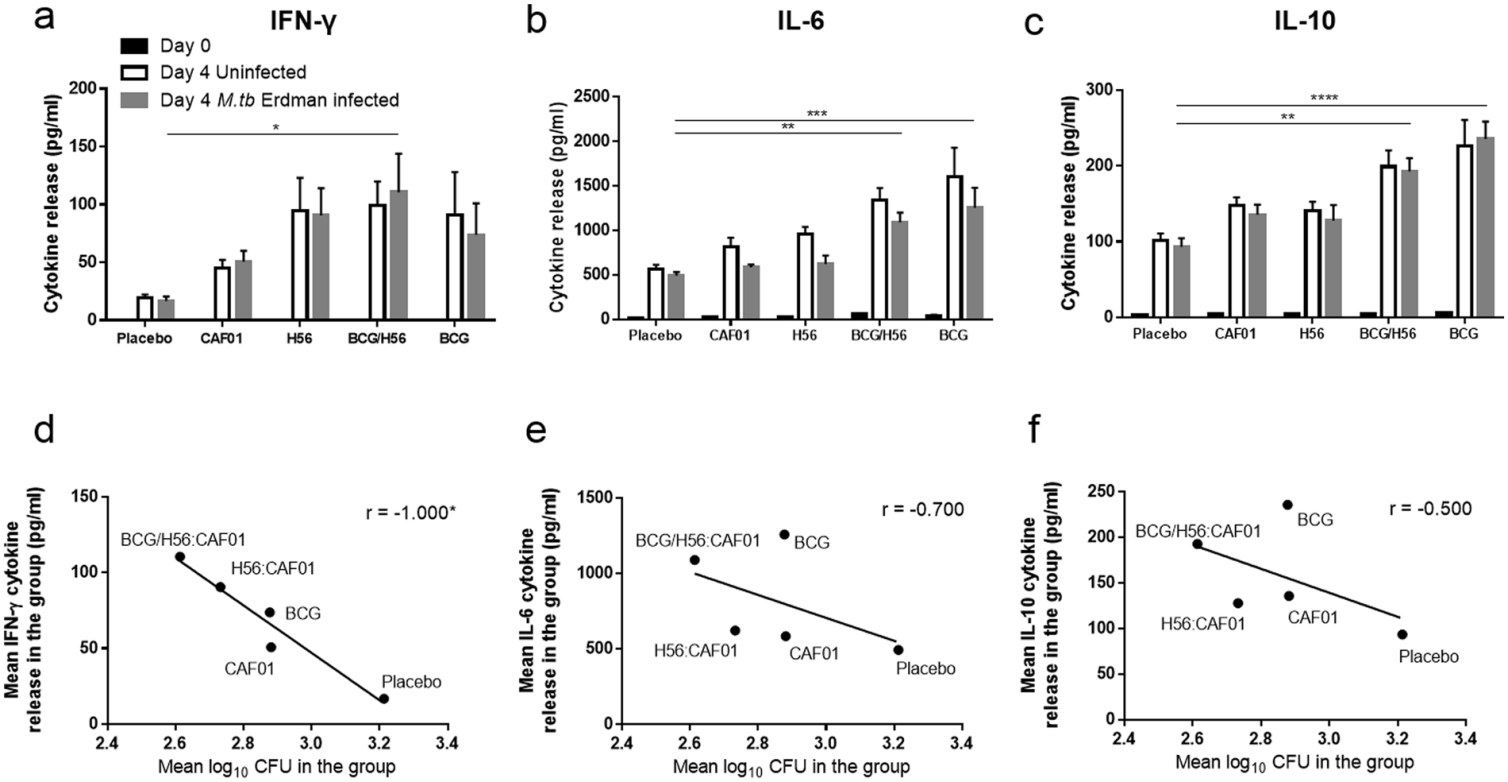
Cytokine release associated with vaccination but not infection. Groups of mice were immunised three times s.c. with 2-week intervals with H56 in CAF01, H56:CAF01 SBS with BCG or given placebo (Tris buffer) or CAF01 as controls. At the same time, as the first vaccination, a group of mice received a single dose BCG. Splenocytes were isolated one week after the last immunisation and used in the MGIA. Culture supernatants were analysed for the released cytokines IFN-γ (**a**), IL-6 (**b**) and IL-10 (**c**). Black bars indicate the levels of cytokines released from splenocytes before *in vitro* culture, while grey bars represent the levels of cytokines measured in the MGIA cultures after four days infection and white bars represent cytokines measured in cultures without infection. Bars represent mean + SEM of eight mice (CAF01 n = 10). For the groups of mice where growth inhibition and MSD data was available (n = 5), scatter plots of mean log_10 _CFU values versus mean levels of IFN-γ (**d**), IL-6 (**e**) and IL-10 (**f**) measured in the same MGIA samples were drawn. One-way ANOVA with Dunnett’s multiple comparisons test was used to compare cytokine levels between vaccinated and placebo control groups (**a–c**). *p < 0.05; **p < 0.01; ***p < 0.001; ****p < 0.0001. (**d–f**) Spearman’s rank *p < 0.05.

As we identified no detectable infection driven expansion of vaccine-specific CD4^+^ T cell populations during the four day culture (Fig. 6), we proceeded to investigate whether we could detect infection specific cytokine response (IFN-γ, IL-1β, IL-6, IL-10, IL-12p70 and TNF-α) in the culture supernatant. Of note, we observed differences in the magnitude of cytokine release between the vaccines, with BCG containing combinations driving the highest levels; in particular H56:CAF01 SBS with BCG immunisation primed significant IFN-γ, IL-6, IL-10 release compared to placebo while BCG immunisation induced significant IL-6 and IL-10 responses (Fig. 8a,b and c (grey bars)). IL-1β and IL-12p70 expression followed the same pattern as IFN-γ, IL-10 and IL-6 however, levels were low (<30 pg/ml) (data not shown). There were no vaccine-specific differences in the magnitude of TNF-α release (stable between 55–60 pg/ml for all vaccines). As suggested by the flow cytometry data earlier, *M.tb* infection did not induce a difference in cytokine responses in any of the investigated vaccine groups (Fig. 8a,b and c (grey bars)).

Next, we explored the association between vaccine-primed cytokine release during four-day *M.tb* splenocyte co-culture and the observed growth inhibition by correlating the mean level of cytokine release and mean growth inhibition in the same group. There was strong significant inverse correlation between IFN-γ release and log_10 _CFU (Spearman r value = −1.0; p = 0.02; Fig. 8d) but not between IL-6 or IL-10 and log_10 _CFU (Spearman r value = −0.7; p = 0.2; Fig. 8e, Spearman r value = −0.5; p = 0.5; Fig. 8f).

## Discussion

In this project, we optimised a reproducible murine splenocyte MGIA using virulent *M.tb* Erdman as the target bacteria. Poor splenocyte viability was identified as a problem in the standard protocol, which could be overcome with simple changes in the culture conditions. Using our optimised MGIA protocol, BCG, H56:CAF01 and H56:CAF01 SBS with BCG induced *M.tb* growth inhibition *in vitro* corresponding to the relative *in vivo* protection^20^. Of note, the adjuvant control also mediated significant growth inhibition at the level of BCG. Assuming that an efficacious TB vaccine (at least in part) control *M.tb* through T cells, we explored vaccine-associated CD4^+^ T cell effector functions, but failed to identify a T cell associated mechanism to explain observed growth inhibition. Spontaneous IFN-γ release in the co-culture supernatant correlated with mycobacterial growth inhibition levels, but the cellular source was not identified.

There remains an incomplete understanding of the host factors that determine why some individuals are protected from *M.tb* infection while others fail to contain infection and progress to active TB. The absence of a protective marker has driven the development of MGIA as a potential correlate of protection encompassing a range of immune mechanisms and their complex interactions^6^. It is a heterogeneous field and a diverse range of assays have been proposed for both humans, mice and cattle^6^. Recently, there has been a move towards protocol harmonisation and standardisation in the field, including the publication of a murine MGIA protocol based on direct co-culturing of mouse splenocytes with BCG^13, 15, 18^, which we and others have used as foundation for murine as well as human PBMC based MGIA studies^22^.

In the MGIA, *M.tb* very rapidly infects cells and becomes intracellular, wherefore it has been an overriding aim of this project to describe the health of the splenocytes and in particular the subpopulation of vaccine-specific CD4^+^ T cells potentially capable of mediating intracellular kill or growth inhibition during the four-day co-culture. We initially explored splenocyte survival under the culture conditions described in the standard splenocyte protocol^15, 18^, and were surprised to find that even in the absence of *M.tb* in the culture, there was a substantial, rapid and reproducible splenocyte death; which could be prevented with simple modifications of the assay (no rotation and use of enriched media). Elaborate explorations with manual and automated counting, other rotators, varying rotator speeds, and reproduction in parallel studies using human PBMCs (Holm personal communication) underpin that the shear forces brought on by rotation negatively affects cell survival^23^. It could be speculated that the cells who encounter their relevant antigen would be more prone to survive. However, we found no indication of a relative increase in the number of specific T cells compared to unspecific T cells on day four. To our knowledge, there is no published data demonstrating the benefit of rotation in the MGIA, and until now there is no studies describing cell viability in these assays^13, 15^. These sobering findings raise concern and call for independent confirmation.

In line with other groups^17, 24^, we used the virulent *M.tb* Erdman as the target bacteria in the MGIA. We consider virulent *M.tb* more relevant than BCG as it expresses more vaccine candidate antigens and allow for better comparison to the *in vivo* challenge experiments we used to benchmark the MGIA assay^25, 26^. Under the assumption that vaccine-induced control of mycobacterial growth may be overwhelmed at higher inoculi^8, 15^, we used ~50 CFU *M.tb* Erdman which in our hands had low variability and comparable growth as higher inoculi. This assay was reproducible and had comparable or lower variability compared to similar splenocyte MGIA described in the literature^13–15, 17^.

We and others have assessed the MGIA potential in splenocytes of BCG-vaccinated mice. Recently Zelmer *et al*. compared the ability of splenocytes from BCG Danish (Statens Serum Institut) and BCG Pasteur (Aeras) vaccinated C57BL/6 mice to mediate growth inhibition of the vaccine BCG *in vitro* using the standard rotator based splenocyte MGIA protocol^15^. Of note, both BCG Pasteur and BCG Danish were protective *in vivo*, but only the BCG Pasteur model was capable of mediating growth inhibition *in vitro* (0.7 log_10 _CFU, CV 23%^15^). BCG Pasteur has also proven capable of mediating growth inhibition of *M.tb* Erdman in the more complex BM/SP-MGIA with pre-infected bone marrow derived macrophage target cells in seven-day splenocyte co-culture^17, 24^. In our assay, BCG Danish mediated a significant growth inhibition of 0.3–0.4 log_10 _CFU with a CV <8%, calling for further studies to elucidate whether BCG Pasteur vaccinated mice or a switch from virulent *M.tb* to the slower growing BCG as target organism would mediate a superior growth inhibition in our model^27^.

As in other studies, we demonstrated an association between individual vaccines ability to control growth *in vitro* and protect *in vivo* ^20, 21^ - an essential positive control supporting the concept of MGIA as a correlate of protection. CD4^+^ T cells are fundamental components of both host control and successful vaccination against TB^28–31^, and a central role for CD4^+^ T cell-mediated growth inhibition has previously been demonstrated in the MB/SP-MGIA model^32^. In the standard splenocyte MGIA model^13, 15, 18^, such a link has only been indicated by an upregulated inflammatory mRNA signature^13^, wherefore we attempted to demonstrate it directly. In agreement with the literature, H56:CAF01 immunisation induced a strong polyfunctional CD4^+^ T-cell profile in our study^19^. Vaccine-specific CD4^+^ T cells in H56:CAF01 immunised mice traffic more efficiently to the *M.tb* infected lung than infection-driven responses^31^ and would be a potential correlate to study in this assay. However, in spite of significant growth inhibition, we failed to demonstrate changes in activation, cytokine expression profile or clonal expansion of vaccine-specific CD4^+^ T cells during four-day co-culture with *M.tb*. These findings do not preclude that a T-cell mediated effect could be demonstrated by using a higher MOI or a preculture step as in the BM/SP-MGIA where more antigen should be available for T-cell recognition. However, in our attempt to describe the mechanisms at play under the conditions which can control infection in the assay, the data suggest that the splenocyte based MGIA rely on a growth inhibitory mechanism(s) which is either very subtle requiring more sensitive assays for detection, or that the mechanism is simply independent of vaccine-specific T cells recognising their antigen on infected cells. The latter interpretation echoed by the significant growth inhibition observed in the negative control (CAF01 adjuvant) group combined with the observation that the vaccine-mediated growth inhibition only could be demonstrated early (1 and 5 weeks post vaccination) and not late (29 weeks post vaccination).

The only investigated factor which correlated with growth inhibition was the level of IFN-γ released in the culture supernatant from day zero to day four. Of note, the IFN-γ levels were comparable in the presence and absence of *M.tb* in the culture, suggesting that the IFN-γ levels do not derive from vaccine-specific T cells recognising their relevant antigen. IFN-γ can be induced by other cells e.g. antigen presenting cells, NK cells and/or neutrophils, cell populations which role in this assay remains to be studied.

In conclusion, we have optimised a murine splenocyte MGIA with *M.tb* Erdman as target organism. The association between vaccine-induced *in vitro* growth inhibition and *in vivo* protection suggested that this assay could represent a relevant tool to compare vaccines and study correlates. However, after failing to demonstrate a direct link between vaccine-induced T cells and growth inhibition, we call for caution drawing firm conclusions on vaccine effects using the splenocyte MGIA before the involved mechanisms are better understood.
