# Author Correction: Optimisation of a murine splenocyte mycobacterial growth inhibition assay using virulent *Mycobacterium tuberculosis*

**DOI:** 10.1038/s41598-024-57464-6

**Published:** 2024-03-28

**Authors:** Christina Jensen, Line Lindebo Holm, Erik Svensson, Claus Aagaard, Morten Ruhwald

**Affiliations:** 1 Department of Infectious Disease Immunology, Statens Serum Institut, Copenhagen, Denmark; 2 Copenhagen University Hospitals, Hvidovre, Copenhagen, Denmark; 3 International Reference Laboratory of Mycobacteriology, Statens Serum Institut, Copenhagen, Denmark

Correction to: *Scientific Reports* 10.1038/s41598-017-02116-1, published online 06 June 2017

This Article contains an error. In the Materials and Methods section, the automated cell counter ‘NucleoCounter®’ and the manufacturer ‘ChemoMetec’ were incorrectly given as ‘Nucelocounter™’ and ‘Chemotec’, respectively.

“The cell suspensions were cultured in 2 ml screw cap tubes (Sarstedt) on a 360° tube rotator (Intelli-Mixer Rm-2 L, ELMI) or in a rack at 37 °C for four days. At different time points, the splenocytes were counted with an automatic Nucelocounter^TM^ (Chemotec) or manually using Nigrosine.”

should read:

“The cell suspensions were cultured in 2 ml screw-cap tubes (Sarstedt) on a 360° tube rotator (Intelli-Mixer Rm-2 L, ELMI) or in a rack at 37 °C for four days. At different time points, the splenocytes were counted with an automatic NucleoCounter® (ChemoMetec) or manually using Nigrosine.”

